# 2-(2-Hy­droxy-2-phenyleth­yl)-1-methyl­cyclo­propan-1-ol

**DOI:** 10.1107/S1600536812051768

**Published:** 2013-01-04

**Authors:** Hui Mao, Ya-Wei Tu, Shi-Kun Li, Xiao-Juan Wang, Peng-Peng Wang

**Affiliations:** a College of Chemistry and Life Science, Zhejiang Normal University, Jinhua 321004, Zhejiang, People’s Republic of China

## Abstract

The asymmetric unit of the title compound, C_12_H_16_O_2_, contains two independent mol­ecules in which the dihedral angles between the benzene and cyclo­propane rings are 75.9 (3) and 76.3 (3)°. In the crystal, the mol­ecules are connected by O—H⋯O hydrogen bonds into a three dimensional supra­molecular structure.

## Related literature

For applications of cyclo­propane derivatives, see: Pietruszka (2003 ▶); Helene *et al.* (2003 ▶); Wessjohann *et al.* (2003 ▶); Charette & Marcoux (1995 ▶).
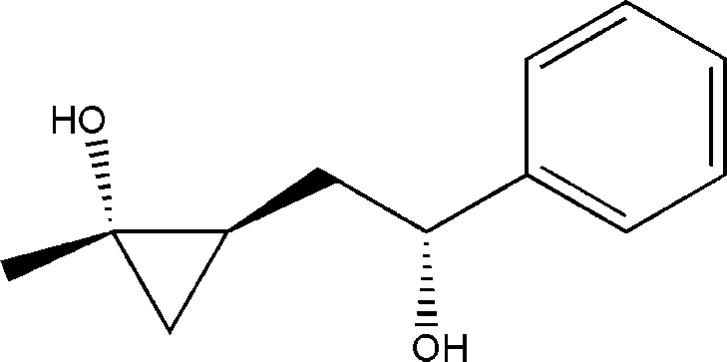


## Experimental

### 

#### Crystal data

C_12_H_16_O_2_
*M* *_r_* = 192.25
Triclinic, 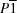

*a* = 9.1700 (8) Å
*b* = 10.3863 (10) Å
*c* = 11.9412 (11) Å
α = 98.133 (7)°
β = 90.854 (6)°
γ = 91.841 (7)°
*V* = 1125.07 (18) Å^3^
*Z* = 4
Mo *K*α radiation
μ = 0.08 mm^−1^
*T* = 296 K
0.13 × 0.10 × 0.08 mm

#### Data collection

Bruker SMART APEXII area-detector diffractometer
16925 measured reflections
5146 independent reflections
2391 reflections with *I* > 2σ(*I*)
*R*_int_ = 0.038

#### Refinement

*R*[*F* ^2^ > 2σ(*F* ^2^)] = 0.070
*wR*(*F* ^2^) = 0.249
*S* = 1.04
5146 reflections
253 parameters
4 restraints
H-atom parameters constrained
Δρ_max_ = 0.37 e Å^−3^
Δρ_min_ = −0.39 e Å^−3^

### 

Data collection: *APEX2* (Bruker, 2006 ▶); cell refinement: *SAINT* (Bruker, 2006 ▶); data reduction: *SAINT*; program(s) used to solve structure: *SHELXS97* (Sheldrick, 2008 ▶); program(s) used to refine structure: *SHELXL97* (Sheldrick, 2008 ▶); molecular graphics: *SHELXTL* (Sheldrick, 2008 ▶); software used to prepare material for publication: *SHELXL97*.

## Supplementary Material

Crystal structure: contains datablock(s) I, global. DOI: 10.1107/S1600536812051768/xu5667sup1.cif

Structure factors: contains datablock(s) I. DOI: 10.1107/S1600536812051768/xu5667Isup2.hkl

Supplementary material file. DOI: 10.1107/S1600536812051768/xu5667Isup3.cml

Additional supplementary materials:  crystallographic information; 3D view; checkCIF report

## Figures and Tables

**Table 1:** Hydrogen-bond geometry (Å, °)

*D*—H⋯*A*	*D*—H	H⋯*A*	*D*⋯*A*	*D*—H⋯*A*
O1—H1*A*⋯O2^i^	0.82	1.94	2.745 (2)	167
O1*A*—H1*AA*⋯O2*A* ^ii^	0.82	1.95	2.757 (2)	167
O2—H2*B*⋯O1*A*	0.82	1.96	2.768 (3)	167
O2*A*—H2*AB*⋯O1^iii^	0.82	1.98	2.778 (2)	165

Symmetry codes: (i) 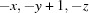; (ii) 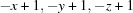; (iii) 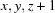.

**Fig. 1.**
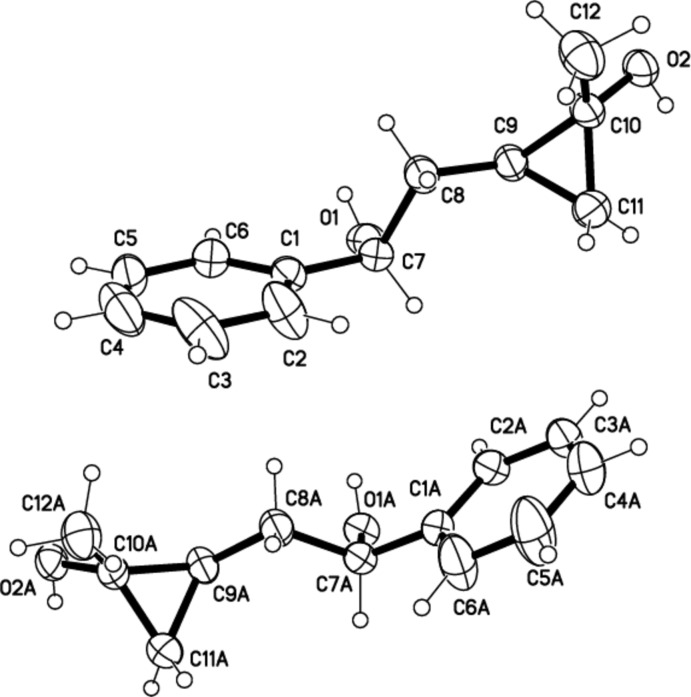
A view of the molecule of (I) showing the atom-labelling scheme with displacement ellipsoids drawn at the 30% probability.

## supplementary crystallographic information

### Comment

Due to the special structure and versatile biologically activity of the chiral cyclopropanes their medicinal properties and synthetic utility have inspired numerous chemists to fascinate (Pietruszka, 2003; Helene *et al.*, 2003; Wessjohann *et al.*, 2003; Charette & Marcoux, 1995). In this work, we reported the synthesis and crystal structure of *cis*-2-(2-hydroxy-2-phenyl-ethyl)-1-methyl-cyclopropanol.

X-ray crystallography confirmed the molecular structure and the atom connectivity for the title compound(I), as illustrated in Fig. 1. A view on the crystal structure of the title compound, the angle of (C9—C10—C11) is 60.8 (2)°, and the angle of (C10—C9—C11) is 59.4 (2)°, and the angle of (C9—C11—C10) is 59.8 (2)°. It can be speculated that the structure of the three ring was similar equilateral triangle. The dihedral angle between the benzene ring and the cyclopropane ring is 75.9 (3) and 76.3 (3)°. The structure is more stable by intramolecular hydrogen bond (O2—H2B···O1A). The intermolecular hydrogen (O1A—H1AA···O2A; O1—H1A0···O2; O2A—H2AB···O1) results in the formation of a three-dimensional structure in the crystal.

### Experimental

To a two-necked flask containing samarium powder (2.5 mmol), was added THF (18 ml) and ally bromide (2.2 mmol) under nitrogen. The mixture was allowed to stir at room temperature for 1 h (the color would turn into purple). HMPA (2.0 ml) and H_2_O (1.0 mmol) was then added in sequence *via* a syringe. A solution of 4-Acetoxy-4-phenyl-1-butene (1.0 mmol) in THF (5.0 ml) was subsequently added. The color would fade out in 3 h (monitored by TLC). After treatment, afford the solid products. Recrystallization condition: Petrol/EtOAc (5/1, v:v), room temperature, one day.

### Refinement

H atoms were positioned geometrically and refined using a riding model with C—H = 0.93–0.98 Å and O—H = 0.82 Å, *U*_iso_(H) = 1.2*U*_eq_(C) and 1.5*U*_eq_(O).

### Crystal data

**Table:**

C_12_H_16_O_2_	*Z* = 4
*M**_r_* = 192.25	*F*(000) = 416
Triclinic, *P*1	*D*_x_ = 1.135 Mg m^−^^3^
Hall symbol: -P 1	Mo *K*α radiation, λ = 0.71073 Å
*a* = 9.1700 (8) Å	Cell parameters from 2877 reflections
*b* = 10.3863 (10) Å	θ = 1.7–27.8°
*c* = 11.9412 (11) Å	µ = 0.08 mm^−^^1^
α = 98.133 (7)°	*T* = 296 K
β = 90.854 (6)°	Block, colourless
γ = 91.841 (7)°	0.13 × 0.10 × 0.08 mm
*V* = 1125.07 (18) Å^3^	

### Data collection

**Table:**

Bruker SMART APEXII area-detector diffractometer	2391 reflections with *I* > 2σ(*I*)
Radiation source: fine-focus sealed tube	*R*_int_ = 0.038
Graphite monochromator	θ_max_ = 27.8°, θ_min_ = 1.7°
ω scans	*h* = −11→11
16925 measured reflections	*k* = −13→13
5146 independent reflections	*l* = −15→15

### Refinement

**Table:**

Refinement on *F*^2^	Primary atom site location: structure-invariant direct methods
Least-squares matrix: full	Secondary atom site location: difference Fourier map
*R*[*F*^2^ > 2σ(*F*^2^)] = 0.070	Hydrogen site location: inferred from neighbouring sites
*wR*(*F*^2^) = 0.249	H-atom parameters constrained
*S* = 1.04	*w* = 1/[σ^2^(*F*_o_^2^) + (0.1296*P*)^2^ + 0.0141*P*] where *P* = (*F*_o_^2^ + 2*F*_c_^2^)/3
5146 reflections	(Δ/σ)_max_ < 0.001
253 parameters	Δρ_max_ = 0.37 e Å^−^^3^
4 restraints	Δρ_min_ = −0.39 e Å^−^^3^

### Special details

**Table:**

Geometry. All e.s.d.'s (except the e.s.d. in the dihedral angle between two l.s. planes) are estimated using the full covariance matrix. The cell e.s.d.'s are taken into account individually in the estimation of e.s.d.'s in distances, angles and torsion angles; correlations between e.s.d.'s in cell parameters are only used when they are defined by crystal symmetry. An approximate (isotropic) treatment of cell e.s.d.'s is used for estimating e.s.d.'s involving l.s. planes.
Refinement. Refinement of *F*^2^ against ALL reflections. The weighted *R*-factor *wR* and goodness of fit *S* are based on *F*^2^, conventional *R*-factors *R* are based on *F*, with *F* set to zero for negative *F*^2^. The threshold expression of *F*^2^ > σ(*F*^2^) is used only for calculating *R*-factors(gt) *etc*. and is not relevant to the choice of reflections for refinement. *R*-factors based on *F*^2^ are statistically about twice as large as those based on *F*, and *R*- factors based on ALL data will be even larger.

### Fractional atomic coordinates and isotropic or equivalent isotropic displacement parameters (Å^2^)

**Table:**

	*x*	*y*	*z*	*U*_iso_*/*U*_eq_	
O1A	0.29470 (19)	0.48083 (17)	0.31228 (15)	0.0595 (5)	
H1AA	0.3789	0.5069	0.3051	0.089*	
O1	0.20635 (19)	0.51624 (17)	−0.18527 (15)	0.0608 (6)	
H1A	0.1211	0.4912	−0.1976	0.091*	
O2A	0.44023 (19)	0.39198 (17)	0.70678 (14)	0.0584 (5)	
H2AB	0.3622	0.4207	0.7296	0.088*	
O2	0.06177 (18)	0.60637 (17)	0.23173 (14)	0.0602 (5)	
H2B	0.1389	0.5758	0.2490	0.090*	
C1	0.1876 (3)	0.7210 (3)	−0.2628 (2)	0.0542 (7)	
C1A	0.3172 (3)	0.2795 (3)	0.1811 (2)	0.0536 (7)	
C2A	0.3688 (3)	0.3498 (3)	0.0998 (2)	0.0595 (8)	
H2AA	0.3859	0.4391	0.1177	0.071*	
C2	0.2208 (5)	0.8513 (3)	−0.2580 (3)	0.0933 (12)	
H2A	0.2619	0.8971	−0.1919	0.112*	
C3A	0.3958 (3)	0.2885 (3)	−0.0095 (2)	0.0703 (9)	
H3AA	0.4298	0.3374	−0.0638	0.084*	
C3	0.1944 (5)	0.9163 (4)	−0.3494 (4)	0.1107 (15)	
H3A	0.2164	1.0051	−0.3438	0.133*	
C4A	0.3729 (4)	0.1591 (4)	−0.0366 (3)	0.0859 (10)	
H4AA	0.3920	0.1186	−0.1092	0.103*	
C4	0.1365 (5)	0.8511 (5)	−0.4469 (3)	0.1019 (14)	
H4A	0.1173	0.8949	−0.5080	0.122*	
C5	0.1070 (4)	0.7221 (5)	−0.4549 (3)	0.0865 (11)	
H5A	0.0701	0.6768	−0.5227	0.104*	
C5A	0.3223 (6)	0.0882 (4)	0.0417 (3)	0.1164 (15)	
H5AA	0.3077	−0.0013	0.0230	0.140*	
C6	0.1309 (3)	0.6553 (3)	−0.3632 (3)	0.0684 (8)	
H6A	0.1085	0.5665	−0.3697	0.082*	
C6A	0.2918 (5)	0.1479 (3)	0.1504 (3)	0.0943 (12)	
H6AA	0.2539	0.0982	0.2028	0.113*	
C7A	0.2894 (3)	0.3416 (3)	0.3008 (2)	0.0536 (7)	
H7AA	0.1916	0.3131	0.3213	0.064*	
C7	0.2137 (3)	0.6552 (2)	−0.1590 (2)	0.0509 (7)	
H7A	0.3115	0.6818	−0.1283	0.061*	
C8	0.1041 (3)	0.6937 (3)	−0.0678 (2)	0.0555 (7)	
H8A	0.1025	0.7879	−0.0521	0.067*	
H8B	0.0076	0.6617	−0.0949	0.067*	
C8A	0.3980 (3)	0.3031 (3)	0.3855 (2)	0.0578 (7)	
H8AA	0.4006	0.2089	0.3771	0.069*	
H8AB	0.4944	0.3362	0.3694	0.069*	
C9	0.1402 (3)	0.6401 (3)	0.0402 (2)	0.0533 (7)	
H9A	0.1507	0.5456	0.0304	0.064*	
C9A	0.3607 (3)	0.3547 (3)	0.5063 (2)	0.0516 (7)	
H9AA	0.3466	0.4487	0.5197	0.062*	
C10A	0.4153 (3)	0.3000 (3)	0.6074 (2)	0.0517 (7)	
C10	0.0892 (3)	0.6972 (3)	0.1554 (2)	0.0533 (7)	
C11A	0.2598 (3)	0.2770 (3)	0.5716 (2)	0.0624 (8)	
H11A	0.1871	0.3236	0.6179	0.075*	
H11B	0.2291	0.1901	0.5369	0.075*	
C11	0.2449 (3)	0.7152 (3)	0.1265 (2)	0.0629 (8)	
H11C	0.3163	0.6664	0.1622	0.076*	
H11D	0.2786	0.8012	0.1136	0.076*	
C12A	0.5203 (3)	0.1925 (3)	0.6002 (3)	0.0743 (9)	
H12A	0.5398	0.1715	0.6746	0.111*	
H12B	0.6096	0.2195	0.5682	0.111*	
H12C	0.4789	0.1171	0.5531	0.111*	
C12	−0.0105 (4)	0.8086 (3)	0.1728 (3)	0.0788 (10)	
H12D	−0.0290	0.8303	0.2520	0.118*	
H12E	−0.1010	0.7847	0.1325	0.118*	
H12F	0.0341	0.8825	0.1450	0.118*	

### Atomic displacement parameters (Å^2^)

**Table:**

	*U*^11^	*U*^22^	*U*^33^	*U*^12^	*U*^13^	*U*^23^
O1A	0.0571 (12)	0.0643 (13)	0.0578 (12)	0.0002 (9)	0.0051 (9)	0.0112 (9)
O1	0.0495 (11)	0.0673 (13)	0.0670 (13)	0.0025 (9)	0.0076 (9)	0.0137 (10)
O2A	0.0563 (11)	0.0765 (13)	0.0422 (10)	0.0118 (9)	0.0067 (8)	0.0049 (9)
O2	0.0571 (12)	0.0786 (13)	0.0495 (11)	0.0111 (9)	0.0044 (9)	0.0220 (9)
C1	0.0516 (16)	0.0657 (19)	0.0465 (16)	0.0064 (13)	0.0071 (12)	0.0103 (13)
C1A	0.0501 (16)	0.0640 (18)	0.0474 (16)	−0.0004 (13)	−0.0040 (12)	0.0119 (13)
C2A	0.0547 (17)	0.0686 (19)	0.0565 (18)	0.0020 (13)	0.0050 (13)	0.0123 (15)
C2	0.150 (4)	0.075 (2)	0.054 (2)	−0.007 (2)	0.020 (2)	0.0081 (17)
C3A	0.066 (2)	0.096 (3)	0.0518 (19)	0.0038 (17)	0.0051 (15)	0.0182 (17)
C3	0.173 (4)	0.085 (3)	0.084 (3)	0.024 (3)	0.053 (3)	0.038 (2)
C4A	0.112 (3)	0.091 (3)	0.0513 (19)	0.006 (2)	−0.0002 (18)	0.0003 (19)
C4	0.114 (3)	0.134 (4)	0.072 (3)	0.051 (3)	0.034 (2)	0.051 (3)
C5	0.071 (2)	0.143 (4)	0.050 (2)	0.019 (2)	0.0030 (15)	0.025 (2)
C5A	0.214 (5)	0.068 (2)	0.062 (2)	−0.015 (3)	0.000 (3)	−0.0021 (19)
C6	0.0599 (19)	0.088 (2)	0.0585 (19)	0.0036 (15)	0.0003 (15)	0.0134 (17)
C6A	0.151 (4)	0.079 (2)	0.052 (2)	−0.020 (2)	−0.002 (2)	0.0153 (17)
C7A	0.0474 (16)	0.0662 (19)	0.0490 (16)	0.0004 (13)	0.0035 (12)	0.0150 (13)
C7	0.0453 (15)	0.0594 (18)	0.0483 (16)	0.0017 (12)	0.0027 (12)	0.0084 (13)
C8	0.0578 (17)	0.0645 (18)	0.0454 (15)	0.0072 (13)	0.0040 (12)	0.0099 (13)
C8A	0.0610 (18)	0.0658 (18)	0.0482 (16)	0.0062 (13)	0.0042 (13)	0.0125 (13)
C9	0.0543 (16)	0.0615 (18)	0.0450 (16)	0.0095 (13)	0.0033 (12)	0.0091 (13)
C9A	0.0588 (17)	0.0554 (17)	0.0426 (15)	0.0105 (12)	0.0059 (12)	0.0113 (12)
C10A	0.0540 (17)	0.0596 (17)	0.0419 (15)	0.0037 (13)	0.0035 (12)	0.0086 (12)
C10	0.0528 (16)	0.0646 (18)	0.0453 (15)	0.0076 (13)	0.0060 (12)	0.0153 (13)
C11A	0.0568 (18)	0.079 (2)	0.0505 (17)	−0.0056 (14)	0.0066 (13)	0.0086 (14)
C11	0.0570 (18)	0.074 (2)	0.0608 (18)	−0.0034 (14)	−0.0009 (14)	0.0210 (15)
C12A	0.089 (2)	0.072 (2)	0.0641 (19)	0.0181 (17)	0.0008 (16)	0.0132 (16)
C12	0.095 (3)	0.080 (2)	0.066 (2)	0.0279 (18)	0.0148 (18)	0.0174 (17)

### Geometric parameters (Å, º)

**Table:**

O1A—C7A	1.433 (3)	C6A—H6AA	0.9300
O1A—H1AA	0.8200	C7A—C8A	1.511 (4)
O1—C7	1.432 (3)	C7A—H7AA	0.9800
O1—H1A	0.8200	C7—C8	1.514 (3)
O2A—C10A	1.425 (3)	C7—H7A	0.9800
O2A—H2AB	0.8200	C8—C9	1.512 (4)
O2—C10	1.421 (3)	C8—H8A	0.9700
O2—H2B	0.8200	C8—H8B	0.9700
C1—C2	1.371 (4)	C8A—C9A	1.514 (3)
C1—C6	1.379 (4)	C8A—H8AA	0.9700
C1—C7	1.517 (4)	C8A—H8AB	0.9700
C1A—C6A	1.376 (4)	C9—C10	1.507 (3)
C1A—C2A	1.375 (4)	C9—C11	1.512 (4)
C1A—C7A	1.511 (4)	C9—H9A	0.9800
C2A—C3A	1.398 (4)	C9A—C10A	1.491 (3)
C2A—H2AA	0.9300	C9A—C11A	1.506 (4)
C2—C3	1.385 (5)	C9A—H9AA	0.9800
C2—H2A	0.9300	C10A—C11A	1.484 (4)
C3A—C4A	1.346 (4)	C10A—C12A	1.493 (4)
C3A—H3AA	0.9300	C10—C11	1.485 (4)
C3—C4	1.354 (6)	C10—C12	1.491 (4)
C3—H3A	0.9300	C11A—H11A	0.9700
C4A—C5A	1.347 (5)	C11A—H11B	0.9700
C4A—H4AA	0.9300	C11—H11C	0.9700
C4—C5	1.348 (5)	C11—H11D	0.9700
C4—H4A	0.9300	C12A—H12A	0.9600
C5—C6	1.395 (4)	C12A—H12B	0.9600
C5—H5A	0.9300	C12A—H12C	0.9600
C5A—C6A	1.394 (5)	C12—H12D	0.9600
C5A—H5AA	0.9300	C12—H12E	0.9600
C6—H6A	0.9300	C12—H12F	0.9600
			
C7A—O1A—H1AA	109.5	C7—C8—H8B	109.2
C7—O1—H1A	109.5	H8A—C8—H8B	107.9
C10A—O2A—H2AB	109.5	C9A—C8A—C7A	112.4 (2)
C10—O2—H2B	109.5	C9A—C8A—H8AA	109.1
C2—C1—C6	117.7 (3)	C7A—C8A—H8AA	109.1
C2—C1—C7	119.5 (3)	C9A—C8A—H8AB	109.1
C6—C1—C7	122.8 (3)	C7A—C8A—H8AB	109.1
C6A—C1A—C2A	117.6 (3)	H8AA—C8A—H8AB	107.9
C6A—C1A—C7A	120.0 (2)	C10—C9—C11	58.94 (17)
C2A—C1A—C7A	122.4 (3)	C10—C9—C8	124.1 (2)
C1A—C2A—C3A	120.8 (3)	C11—C9—C8	119.8 (2)
C1A—C2A—H2AA	119.6	C10—C9—H9A	114.3
C3A—C2A—H2AA	119.6	C11—C9—H9A	114.3
C1—C2—C3	121.5 (4)	C8—C9—H9A	114.3
C1—C2—H2A	119.3	C10A—C9A—C8A	124.3 (2)
C3—C2—H2A	119.3	C10A—C9A—C11A	59.36 (17)
C4A—C3A—C2A	120.4 (3)	C8A—C9A—C11A	120.2 (2)
C4A—C3A—H3AA	119.8	C10A—C9A—H9AA	114.1
C2A—C3A—H3AA	119.8	C8A—C9A—H9AA	114.1
C4—C3—C2	120.2 (4)	C11A—C9A—H9AA	114.1
C4—C3—H3A	119.9	O2A—C10A—C9A	115.5 (2)
C2—C3—H3A	119.9	O2A—C10A—C11A	114.9 (2)
C3A—C4A—C5A	119.9 (3)	C9A—C10A—C11A	60.82 (18)
C3A—C4A—H4AA	120.0	O2A—C10A—C12A	111.7 (2)
C5A—C4A—H4AA	120.0	C9A—C10A—C12A	123.1 (2)
C5—C4—C3	119.5 (4)	C11A—C10A—C12A	122.1 (2)
C5—C4—H4A	120.3	O2—C10—C11	115.1 (2)
C3—C4—H4A	120.3	O2—C10—C12	112.2 (2)
C4—C5—C6	121.2 (3)	C11—C10—C12	121.8 (2)
C4—C5—H5A	119.4	O2—C10—C9	115.5 (2)
C6—C5—H5A	119.4	C11—C10—C9	60.72 (17)
C4A—C5A—C6A	120.6 (3)	C12—C10—C9	122.7 (2)
C4A—C5A—H5AA	119.7	C10A—C11A—C9A	59.82 (16)
C6A—C5A—H5AA	119.7	C10A—C11A—H11A	117.8
C1—C6—C5	119.9 (3)	C9A—C11A—H11A	117.8
C1—C6—H6A	120.0	C10A—C11A—H11B	117.8
C5—C6—H6A	120.0	C9A—C11A—H11B	117.8
C1A—C6A—C5A	120.7 (3)	H11A—C11A—H11B	114.9
C1A—C6A—H6AA	119.6	C10—C11—C9	60.34 (17)
C5A—C6A—H6AA	119.6	C10—C11—H11C	117.7
O1A—C7A—C1A	112.3 (2)	C9—C11—H11C	117.7
O1A—C7A—C8A	107.1 (2)	C10—C11—H11D	117.7
C1A—C7A—C8A	112.7 (2)	C9—C11—H11D	117.7
O1A—C7A—H7AA	108.2	H11C—C11—H11D	114.9
C1A—C7A—H7AA	108.2	C10A—C12A—H12A	109.5
C8A—C7A—H7AA	108.2	C10A—C12A—H12B	109.5
O1—C7—C1	112.1 (2)	H12A—C12A—H12B	109.5
O1—C7—C8	107.8 (2)	C10A—C12A—H12C	109.5
C1—C7—C8	112.0 (2)	H12A—C12A—H12C	109.5
O1—C7—H7A	108.3	H12B—C12A—H12C	109.5
C1—C7—H7A	108.3	C10—C12—H12D	109.5
C8—C7—H7A	108.3	C10—C12—H12E	109.5
C9—C8—C7	111.9 (2)	H12D—C12—H12E	109.5
C9—C8—H8A	109.2	C10—C12—H12F	109.5
C7—C8—H8A	109.2	H12D—C12—H12F	109.5
C9—C8—H8B	109.2	H12E—C12—H12F	109.5
			
C6A—C1A—C2A—C3A	−0.8 (4)	O1—C7—C8—C9	62.7 (3)
C7A—C1A—C2A—C3A	178.8 (2)	C1—C7—C8—C9	−173.6 (2)
C6—C1—C2—C3	1.9 (5)	O1A—C7A—C8A—C9A	61.7 (3)
C7—C1—C2—C3	−177.8 (3)	C1A—C7A—C8A—C9A	−174.3 (2)
C1A—C2A—C3A—C4A	−0.6 (4)	C7—C8—C9—C10	157.0 (2)
C1—C2—C3—C4	−1.0 (6)	C7—C8—C9—C11	86.3 (3)
C2A—C3A—C4A—C5A	0.6 (5)	C7A—C8A—C9A—C10A	159.3 (2)
C2—C3—C4—C5	−1.0 (6)	C7A—C8A—C9A—C11A	87.8 (3)
C3—C4—C5—C6	2.0 (6)	C8A—C9A—C10A—O2A	146.8 (2)
C3A—C4A—C5A—C6A	0.7 (7)	C11A—C9A—C10A—O2A	−105.6 (2)
C2—C1—C6—C5	−1.0 (4)	C8A—C9A—C10A—C11A	−107.6 (3)
C7—C1—C6—C5	178.8 (2)	C8A—C9A—C10A—C12A	3.7 (4)
C4—C5—C6—C1	−1.0 (5)	C11A—C9A—C10A—C12A	111.3 (3)
C2A—C1A—C6A—C5A	2.1 (5)	C11—C9—C10—O2	−105.8 (2)
C7A—C1A—C6A—C5A	−177.5 (4)	C8—C9—C10—O2	147.2 (2)
C4A—C5A—C6A—C1A	−2.2 (7)	C8—C9—C10—C11	−107.1 (3)
C6A—C1A—C7A—O1A	−168.9 (3)	C11—C9—C10—C12	111.0 (3)
C2A—C1A—C7A—O1A	11.5 (4)	C8—C9—C10—C12	3.9 (4)
C6A—C1A—C7A—C8A	70.0 (4)	O2A—C10A—C11A—C9A	106.5 (2)
C2A—C1A—C7A—C8A	−109.6 (3)	C12A—C10A—C11A—C9A	−112.8 (3)
C2—C1—C7—O1	−166.3 (3)	C8A—C9A—C11A—C10A	114.4 (3)
C6—C1—C7—O1	13.9 (3)	O2—C10—C11—C9	106.4 (2)
C2—C1—C7—C8	72.5 (3)	C12—C10—C11—C9	−112.3 (3)
C6—C1—C7—C8	−107.3 (3)	C8—C9—C11—C10	114.1 (3)

### Hydrogen-bond geometry (Å, º)

**Table:**

*D*—H···*A*	*D*—H	H···*A*	*D*···*A*	*D*—H···*A*
O1—H1*A*···O2^i^	0.82	1.94	2.745 (2)	167
O1*A*—H1*AA*···O2*A*^ii^	0.82	1.95	2.757 (2)	167
O2—H2*B*···O1*A*	0.82	1.96	2.768 (3)	167
O2*A*—H2*AB*···O1^iii^	0.82	1.98	2.778 (2)	165

Symmetry codes: (i) −*x*, −*y*+1, −*z*; (ii) −*x*+1, −*y*+1, −*z*+1; (iii) *x*, *y*, *z*+1.
